# Supplementary figures and images for: Rhodosporidium toruloides: a new platform organism for conversion of lignocellulose into terpene biofuels and bioproducts
Source: Biotechnol Biofuels. 2017 Oct 23;10:241. doi: 10.1186/s13068-017-0927-5 (PMC5651578; doi:10.1186/s13068-017-0927-5)

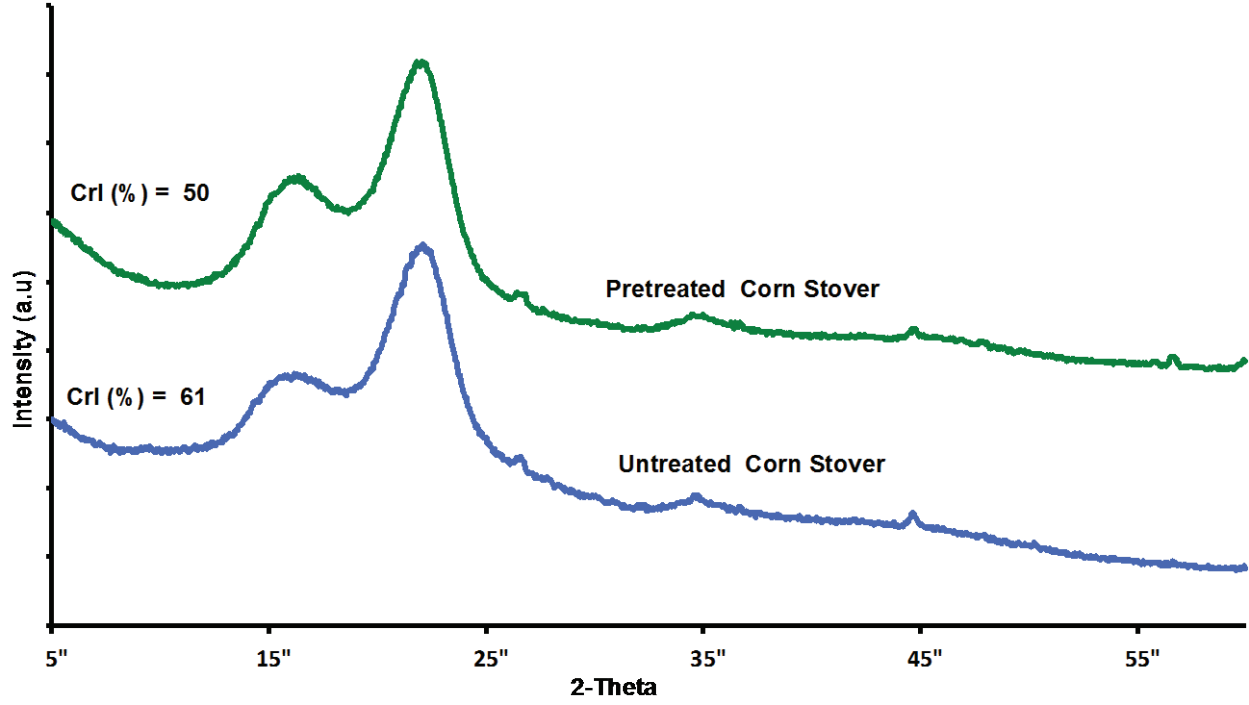

Supplement: Supplementary file 1 — Additional file 1. X-ray diffraction patterns and CrI (%) values of corn stover, both untreated and pretreated with [Ch][α-Kg] (40 wt% in H2O) at 120 °C for 4 h pretreatment condition. [file 13068_2017_927_MOESM1_ESM.pdf]

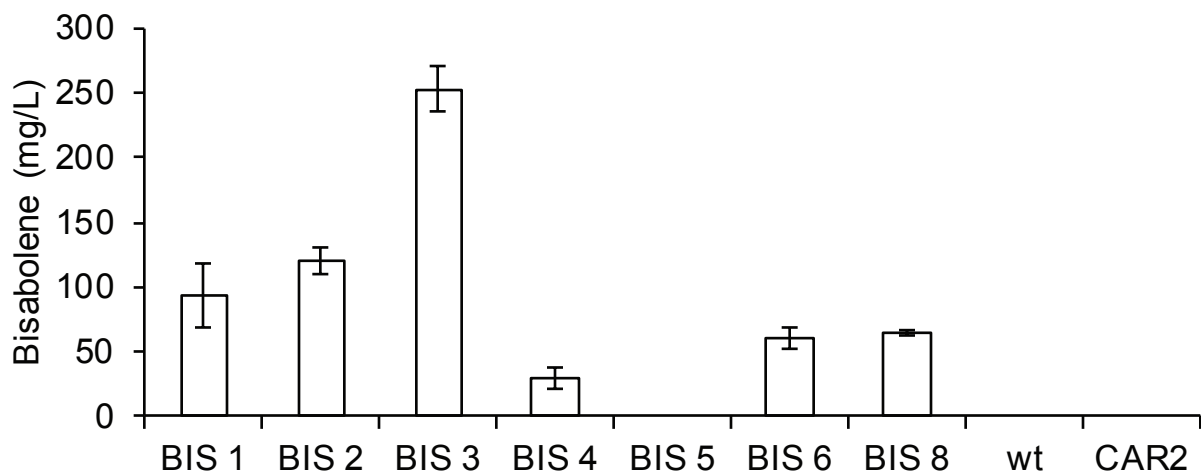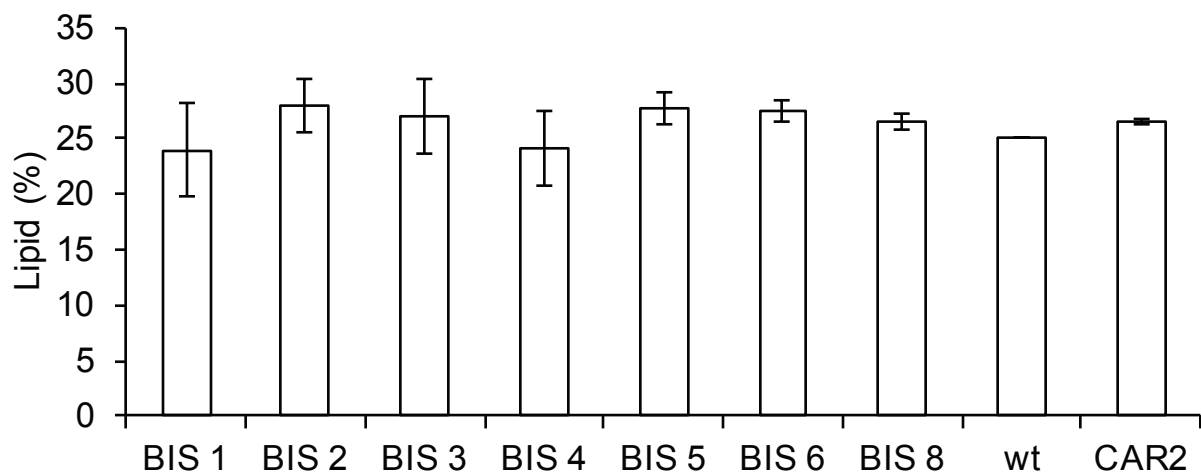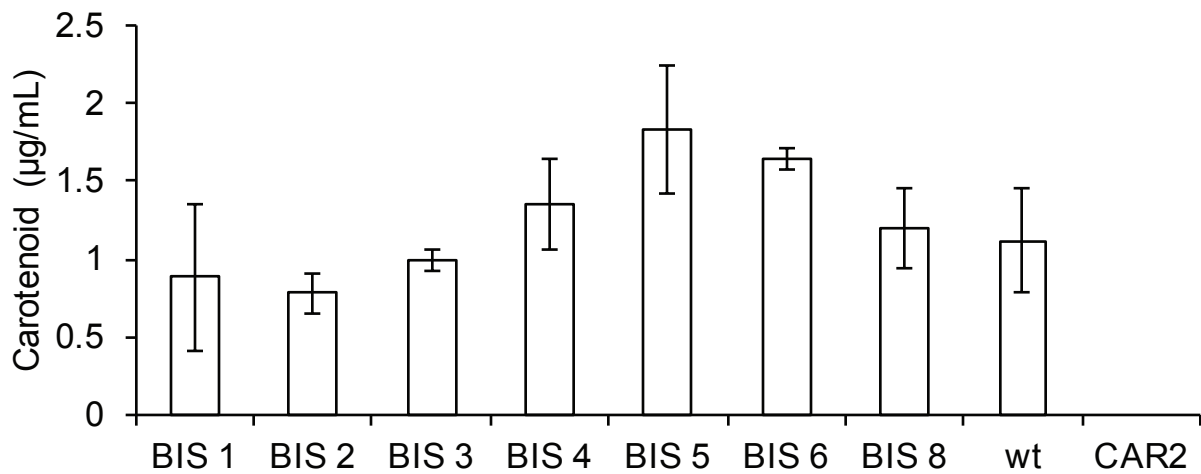

Supplement: Supplementary file 2 — Additional file 2. Comparison of bisabolene titers, lipid content, and carotenoid levels between different BIS transformants. CAR2 refers to a strain harboring a deletion of the phytoene synthase/lycopene cyclase gene and is shown here to demonstrate that lack of carotenoids does not affect lipid production [17]. (n = 3, data shown as average ± standard deviation, from a single experiment). [file 13068_2017_927_MOESM2_ESM.pdf]
